# Four Letters with Replies

**Published:** 1877-01

**Authors:** 


### Our Correspondence.

PENN YAN, N. Y., Dec. 1, 1876.

DR. UP DE GRAFF :

What is good for croup? Many children are dying here from this cause. If you can give us any hints as to its successful management, you will do us a merciful kindness. MRS. W.

Many mistakes are made in the care of children suffering from croup. The sick room is usually kept too warm, doors and windows closed, while every crevice and cranny capable of admitting a breath of fresh air, is carefully caulked. This is the most serious error. The apartment should be as large as possible, the temperature never above 65°, with a window open at the top—the furthest one from the patient's bed—and kept open.

If membrane is forming or *has* formed in the throat, place the little patient in its crib, throw a heavy blanket over the bed, allowing it to rest upon the posts, in such a manner as to cause an enclosed space in which the little sufferer lies. Now, place a lump of unslacked lime—as large as your two fists—in a basin of hot water. Set this at the foot of the bed, being careful not to have it touch the child's limbs, as it will become very hot. While the lime is slacking, keep the blanket around the crib in such a manner as to retain the escaping steam and fumes about the child, so that in his cries and struggles, he is forced to breathe them. Keep your hand under the covering, near the child's face, and when the steam becomes unbearable to your hand, quickly raise the covering in order to admit air. In a moment, confine the vapors again and continue the slacking of the lime for fifteen minutes, regulating the temperature in the manner described. Every two or three hours, repeat the process.

Brush the throat, after every fumigation, with *tincture muriate of iron.* For a child under three years of age, dilute the iron one-half with water. Give three drops of the tincture mentioned, on a lump of sugar, to the child, every four hours, if under three years of age. If from four to six years of age, give five drops. Let the patient have plenty of ice—broken into little pellets and swallowed whole. This they will eat greedily. Remember—*give them all they want.* About the neck, bind a piece of thinly-sliced, salt, fat, pickled pork—"an old woman's remedy," you will remark, but put it there. Feed the child beef-tea and raw egg and wine, moderately enough to preserve its strength. Of course, all this should be carefully performed under the direction of the family physician, who will vary it to suit the case.

We have seen many children saved from impending death by means of the treatment indicated.

---

TOLEDO, O., Nov. 26, 1876.

EDITOR BISTOURY :

I have what my physician calls a loose cartilage in my knee. It gets out of place suddenly, while walking, and lays me up for several weeks, it being utterly impossible for me to touch my foot to the floor. The knee sometimes swells and becomes very painful. Now, what I want to know is, can my trouble be cured? J. R. D.

Apply to some capable surgeon and have the offending cartilage removed. The operation is a safe one and will result in a perfect cure.

---

BLOSSBURG, Pa., Oct. 24, 1876.

DR. UP DE GRAFF:

A traveling optician has been in this place selling spectacles and representing himself as being an agent of yours. I told him I thought he was an impostor. Am I right? S. T. M.

These spectacle venders are becoming nuisances to us. Scarcely a week passes that does not bring an inquiry relative to some chap who represents himself as an agent of ours, or in some manner connected with our establishment. We have repeatedly warned our readers against them, and again declare, once for all, that we never send out agents of any sort, neither do we deal in spectacles.

---

The following letter explains itself:

ALBANY, Ind., May 17, 1876.

ED. BISTOURY:

*Dear Sir:*—I have been using Bromide Potassium in the treatment of epilepsy for several years. The following is the formula that I use, viz:

℞

Potassa Brom., ℥ iv.
Amonia Brom., ℥ ss.
Tinct. Opii, ℥ ss.
Fld. Ex. Ladies Slipper ℥ ij.
Aqua Pura, f ℥ xviij.

Mix.

Sig. A tablespoonful three times a day, and if the case is a bad one, give a dose at bed-time.

I hope that you will give the above room in your valuable journal, so that the profession may give it a fair trial. I also use the same prescription in all nervous troubles of hysterical females, and find it a good thing. Hoping that others may receive the same benefit from the above, I am

Yours, &c.,

A. P. MURRAY, M. D.

